# Psychiatric Advance Directives and Artificial Intelligence: A Conceptual Framework for Theoretical and Ethical Principles

**DOI:** 10.3389/fpsyt.2020.622506

**Published:** 2021-01-22

**Authors:** Stéphane Mouchabac, Vladimir Adrien, Clara Falala-Séchet, Olivier Bonnot, Redwan Maatoug, Bruno Millet, Charles-Siegfried Peretti, Alexis Bourla, Florian Ferreri

**Affiliations:** ^1^Sorbonne Université, AP-HP Department of Psychiatry, Hôpital Saint-Antoine, Paris, France; ^2^Sorbonne Université, iCRIN Psychiatry (Infrastructure of Clinical Research In Neurosciences - Psychiatry), Brain and Spine Institute (ICM), INSERM, CNRS, Paris, France; ^3^Laboratory of Psychopathology and Health Processes, EA 4057, Institute of Psychology, University of Paris, Paris, France; ^4^CHU de Nantes, Department of Child and Adolescent Psychiatry, Nantes, France; ^5^Pays de la Loire Psychology Laboratory, EA 4638, Nantes, France; ^6^Sorbonne Université, AP-HP Department of Psychiatry, Hôpital Pitié-Salpêtrière, Paris, France; ^7^Jeanne d'Arc Hospital, INICEA Group, Saint-Mandé, France

**Keywords:** psychiatric advance directives, artificial intelligence, medical ethics, joint crisis plan, clinical decision support system, predictive medicine

## Abstract

The patient's decision-making abilities are often altered in psychiatric disorders. The legal framework of psychiatric advance directives (PADs) has been made to provide care to patients in these situations while respecting their free and informed consent. The implementation of artificial intelligence (AI) within Clinical Decision Support Systems (CDSS) may result in improvements for complex decisions that are often made in situations covered by PADs. Still, it raises theoretical and ethical issues this paper aims to address. First, it goes through every level of possible intervention of AI in the PAD drafting process, beginning with what data sources it could access and if its data processing competencies should be limited, then treating of the opportune moments it should be used and its place in the contractual relationship between each party (patient, caregivers, and trusted person). Second, it focuses on ethical principles and how these principles, whether they are medical principles (autonomy, beneficence, non-maleficence, justice) applied to AI or AI principles (loyalty and vigilance) applied to medicine, should be taken into account in the future of the PAD drafting process. Some general guidelines are proposed in conclusion: AI must remain a decision support system as a partner of each party of the PAD contract; patients should be able to choose a personalized type of AI intervention or no AI intervention at all; they should stay informed, i.e., understand the functioning and relevance of AI thanks to educational programs; finally, a committee should be created for ensuring the principle of vigilance by auditing these new tools in terms of successes, failures, security, and relevance.

## 1. Introduction

### 1.1. Psychiatric Advance Directives (PADs)

Psychiatric disorders are often characterized by a high rate of relapse, during which the patient's decision-making abilities are altered and may result in psychiatric admissions, often involuntarily. Laws usually indicate that patients cannot be treated without their consent. But there is, most often, a legal framework to provide care or hospitalize patients who can no longer give free and informed consent. These patient care modalities are often deemed to be very restrictive, disempowering, and stigmatizing. Mostly, they predicted poorer recovery and more suicidal ideation after 2 years, mediated by decreased empowerment after 1 year. Finally, they do not promote a proper therapeutic relationship between patient and physician (1–3). Furthermore, compulsory admission is often used inappropriately to manage aggressive behavior rather than psychiatric diseases (4). In addition, routine crisis treatment guidelines are developed without patient involvement and based upon standard recommendations that are not personalized.

In most countries, patients have the right to give an informed advance treatment framework [advance directives (ADs)], which allows anticipating their requests for future care and providing information about drug treatments, nonmedical instructions, and the person authorized to make decisions for them (5). For example, in France, the L1111-11 article of the French Public Health Code (FPHC) and in United Kingdom the Mental Capacity Act 2005, amended by the Mental Health Act 2007, frame this practice. On a wider scope, The United Nations Convention on the Rights of Persons with Disabilities encourages the use of strategies that promote patient decision-making autonomy.

Foreseeing such situations is considered as the hallmarks of good clinical practice, recognized as an important supported decision-making tool and it also enables the patient to ensure that the medical decision is most consistent with his own interests. To be valid, the patient must have the mental capacity to write by hand the ADs. “Mental capacity” is defined as the ability to make a decision, understand, and analyze information related to one's care and know the existing alternative options (6).

In a recent review (7), authors clustered PADs into four types depending of their content, the way they are drawn up, and the level of legal authority:

- Classic PADs are formalized by the patient without any caregivers interventions and describes personal values and treatment preferences. They give informed consent to therapeutic interventions (which are accepted or rejected) and name the proxy for decisions during a relapse or crisis.
- Simplified PADs are a form of directive in which trained caregivers helps the user to create the final document, which would increase its quality. Among the methods available, the use of a semi-structured interview allows to select the preferences for future treatments based on a set of available information.
- Cognitive Therapy-based Advance Directives are written with a staff member, taking events from previous crises and proposing alternatives for future episodes (8). Finally, there is a collaborative approach between the patient and the caregiver, in which disagreements and differences of opinion are respected and recognized.
- The Joint Crisis Plan involves the patient and the care team in a negotiation process with a third-party facilitator who may be a mental health worker, a family member, a trusted person, a custodian, or a lawyer, and the quality of the document could be assessed with a “quality of crisis plan” checklist.

In general, the proposal to draft PADs is well-received by patients and health professionals (9, 10). The feeling of having an active participation in the decision-making process (9), the opportunity to record a treatment refusal (10), mutual agreement, and clarification also strengthens the therapeutic alliance and trust between the patient and the care team (11). Patients report a greater perceived sense of control over their care and treatment journey in a potential context of impaired capacity to make appropriate, informed decisions. Indeed, PADs are associated with a benefit such as improving the autonomy of patients and promoting empowerment, which has been defined as “the ongoing capacity of individuals or groups to act on their own behalf to achieve a greater measure of control over their lives” (12). In addition, the drafting of PADs has also shown its relevance in reducing the traumatic and coercive experience of a treatment not chosen by the patient and implemented in an emergency situation. Adherence to care is optimized and there is a decrease in the rate of coercive intervention or hospitalizations in psychiatry compared to consumers without PADs [(13), in a ratio of 50% over a 24-month follow-up period (14). This may be due to both a greater involvement of patients in their care experience and a more detailed understanding of their disorders (9). As expected, PADs may also reduce negative coercive treatment experiences and stigma (15) and is a strong enhancer of therapeutic relationship (16).

This “Advance Statement” also refers to the possibility of identifying prodromal signs of relapse and proposing early personalized interventions. This kind of approach is promising; on the one hand, it allows anticipating the aggravation of the symptoms and the intensity of the relapse. On the other hand, it offers the possibility of choosing treatments by stage more adapted to the patients (16).

Despite this recommendations and level of evidence, some studies have identified barriers to ADs use, which are clustered into health system constraints, health professional practices, and service user representations (17, 18). Predictive medicine has emerged largely in recent decades, including in psychiatry where recent advances in genetics, neuroimaging, and biomarkers are clarifying neurobiological features of mental diseases and could lead to the development of effective personalized medicine (19) and more appropriate ADs. Nevertheless, when we consider the complexity of gene–environment interaction, the use of ADs in psychiatry is complex, even risky. In this field, “to predict” may be understood as the action to announce in advance what should happen by intuition, reasoning, conjecture, or experience. If we retain the scientific aspect of this definition, the possibility of predicting the occurrence of a morbid event opens important perspectives, whether preventive or curative, but also ethical issues.

### 1.2. Artificial Intelligence (AI) Enhanced Clinical Decision Support Systems (CDSS)

The use of CDSS may be a response to this reluctance and is fundamental for proposing “staged” ADs in function of the intensity of the symptoms. Historically, CDSS belong to three registers: Bayesian probabilistic models, score calculations, and expert systems based on syllogistic algorithms. Interestingly, AI technologies and machine learning methods offer attractive prospects to design and manage crisis response processes in the form of new CDSS. Here, we are alluding to “weak AI,” which consists of a device able to learn and correct itself [whereas “strong AI,” i.e., autonomous machines with adaptation capacities, is still far beyond reach (20)]. The main purpose of weak AI is the valorization of human skills that are not possessed or that should not be possessed by AI for ethical reasons or the precautionary principle (21). Many initiatives are made in this direction, for example the recent creation of an international observatory of social impacts of AI and digital technologies (https://observatoire-ia.ulaval.ca).

AI technologies could use the user's digital phenotype on the phone's health data captured continuously (e.g., number of steps) or occasionally. The concept of digital phenotyping appears to be very efficient to implement data for these systems. Introduced by Jain et al. (22), it is based on the idea of collecting in real time human behavior data (the momentary ecological assessment or EMA) and markers of their functioning in order to characterize the “digital signature of pathology.” The emotions, the energy level, or the presence of symptoms with their perceived intensity (ruminations, hallucinations, and suicidal ideation) can be analyzed. These data can provide useful indicators to identify the increased symptomatology (crisis, manic episode) of many pathologies (bipolar disorder, schizophrenia, major depressive episode, substance abuse) such as logorrhea, increased communicability or reduced social contact, increased behavioral activation, agitation, or psychomotor deceleration (23–30). For instance, CDSS enhanced with AI could make compulsory admissions more efficient to provide appropriate psychiatric care (4).

In addition to these data, the increase in the use of mobile and chatbots applications (providing exchange, therapeutic exercises) creates sources of declarative data on the patient's condition that are particularly interesting to better understand what the person is experiencing in their daily lives, to anticipate relapses, and to better treat such disorders. This collection of subjective data is clearly crucial in medicine and in psychiatry in particular. The notion of contextualization is central in order to personalize follow-up and better understand the appearance of symptoms. Since all these data exceeds the psychiatrist's real-time analysis capabilities, many of the difficulties encountered in consultation (forgetfulness, recall bias, loss of valuable context elements) could be overcome in the data obtained through AI technologies. They could exploit this digital signature by confronting it with important databases that group those of other patients to draw predictive information from them.

Digital phenotype also gives useful first points of contact in the detection of a crisis that can be used, if the patient has given his or her consent, to inform the treating health *in situ* professionals. This provides the opportunity to set up an emergency consultation to deal more effectively with the difficulty when it arises.

Overall, AI technologies offer CDSS tools that are interesting in clinical evaluation by creating relevant algorithms for diagnosis, decision support, relapse prediction, and neuro-prediction. Including the patient's consent, data extraction, and anonymization, this could contribute to a certain therapeutic innovation by creating an ample database: phenotypes (typical symptom profiles, relevant indicators) and patterns (monitored treatments, epidemiological data). For instance, in France, “Health Data Hub” is the initiative following these goals. This combination of tools could be useful and could facilitate PAD completion as peers and caregivers already do (18).

### 1.3. Issues in Predictive Medicine

Nevertheless, the issue of complex decisions, as is often the case in situations covered by PADs, raises the question of the impact of a nonhuman making decision: such a decision proposed by a nonhuman entity appears to be safer, more rational than that of a human because it is based on a very large amount of data and algorithms with few margins of error. However, the function of these data leads to the question of why and how to use these data: how often should these data be used, how often should these systems be transmitted to the professional? Based on what criteria? From when and what does an indicator provide valid information about the worsening of a disorder? Is there not a greater risk of overreaction or prediction error? Before implementing AI in the PAD drafting process, we must ask ourselves what and where ethical limits should be drawn. This is the goal of a predictive medicine (31).

The purpose of this conceptual framework is to assess two fundamental dimensions of the implementation of AI in the PAD drafting process. First, we will address the issue of the nature of AI (how it functions and interacts with databases) and its place during the process or in the patient–professional relationship. Second, we will be focusing on the ethical principles that this implementation should respect.

## 2. Levels of AI Intervention in the PAD Drafting Process

AI can intervene in many ways in the PAD drafting process. First of all, the patient should be able to choose if he/she agrees with AI intervention and if he/she does, then he/she should be able to choose in which way.

### 2.1. Various Natures of AI

We can describe user or developer's intentional limitation of AI by separating two different AI functionalities:

#### 2.1.1. “WHAT”—In Terms of Access to Data Sources

Data sources are on different graduated levels, from public available sources (Google search, civil status, etc.) to semi-public (social networks, medical data) and finally private (mailbox, web-browser history, etc.). As especially in psychiatry, useful information is often of a private nature, and the right to privacy should be protected. For PADs, particular attention should be paid to the way in which data are used: explanations to patients of the issues and rights, patients must give their free and informed consent to the use that can be made of their data, what data they wish to have added to a knowledge database or not, and how they can exercise the right to withdraw. Also, it would be appropriate for the professional and the patient to agree on the symptoms to be followed, the key indicators of relapse. The patient can then choose which symptoms will be monitored by the AI, collected, and transmitted to the professional and/or a team of professionals.

#### 2.1.2. “HOW”—In Terms of Different Intelligences (32)

Like conscience (projections of intention, metacognition, etc.), cognition processes of AI can be split, and the patient can be allowed to choose only specific processes, following the recommendation of Villani et al. (21) to preserve certain human skills for humans only. In practice, all these questions are related to the place of AI in care at the time of the crisis. The degree of trust in new technologies of each participant in the PAD drafting process has an impact on the place given to AI. The professional therefore has a major role to play in the way he or she presents technological tools, the patient's understanding of them, the degree of acceptance, and the ability to delegate the decision to a machine. It will be necessary to find the acceptable ratio for everyone between human and nonhuman expertise and ensure that the patient's wishes are respected. This is one of the key points for the drafting of PADs: the possibility offered by the AI, as an expert authority, to ensure that the patient's wishes are respected in terms of type of care.

### 2.2. Various Places of AI

#### 2.2.1. “WHEN”—At Opportune Moments

**Figure 1:**
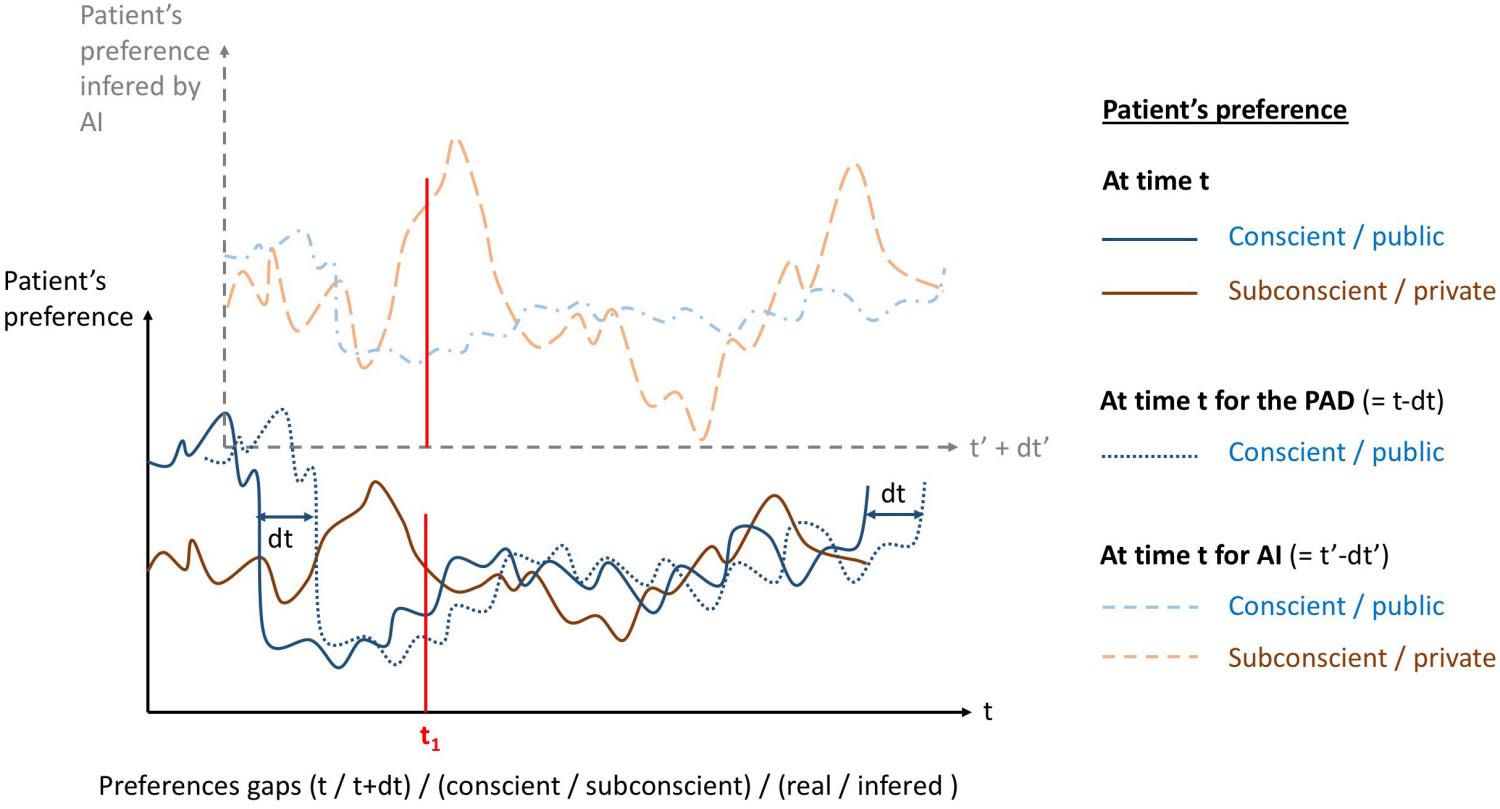
Patient's preference seen as a multivariate function, here represented as a function of time. Real conscient/public (blue line) and subconscient/private (brown line) preferences variate with time. Psychiatric advance directives (PADs) only gives the conscient/public preference but shifted in time (blue dotted line) since there is a time *dt* between the PAD drafting and the moment it applies. Artificial intelligence (AI) implemented to PADs infers conscient/public preference shifted in time but also shifted in nature (dashed blue line) by definition of inference: it do not gives the real preference anymore. AI could also infer subconscient/private preference (dashed brown line).

- *At the moment of the drafting process*: AI could propose a PAD template based on deep learning.
- *To optimize PADs in real-time*: ADs were developed because current care guidelines are either over or under inclusive. AI makes it possible to optimize ADs in real time through an incrementation process. ADs propose a decision that will prevail against a future will. But patients' preference may change overtime, and there is always a shift between their preference at the time *t* and their preference at the previous time *t*−*dt* of the AD: the preference can be seen as a multivariate function of sociodemographic data, environmental factors, and time (Figure 1). For example, if the environmental circumstances change, it is possible for the directives to be modified and adapted to the new context. This kind of microdirectives could be extracted from large databases, but personalized too: from an incremental point of view, these microdirectives could be enhanced with the others patient's experiences after an algorithmic treatment with AI, so new directives benefit from past directives. This feedback permits the selection of a new panel of possible and efficient directives. The use of AI will create a supplementary shift, between the real preference and the inferred preference, independently of the time factor. Furthermore, ADs express conscious or public preference, whereas AI could access the unconscious or private preference, raising the ethical issue of which preference is the most beneficial for the patient (Figure 1). The incrementation process and feedback offer a large field of directives for the constitution of the ADs that could act as “validated alarms” for the different actors implicated in ADs. This principle is already used in law (33). Still, regarding PADs, the issue is made more complex because the clinician must decide whether patients are currently able to express their preference or not: the preference inferred by AI (different of the real preference) could turn to influence this clinical decision and induce an error that could self-drive itself, the patient real preference ending with being rejected permanently.
- *At the moment of a “difficult” medical decision*: The relevance of AI is in particular to help the professional in the event of a difficult decision (34), with complex, contradictory data, and evolution. The use of AI, coupled with ADs—a device also recognized as helping in difficult medical decision-making (35)—would facilitate the actions of professionals in accordance with the patient's request. By difficult decision, we mean in particular the case of a gap between what the patient would have liked (as noted in his/her AD) and therapeutic options, considering the ongoing situation. This can occur in this kind of situation: new information on the patient's situation (whether or not detected by AI), conflict of interest between the clinical benefit of a therapeutic modality and the patient's choices, and new therapeutic modalities available not provided for in advance patient instructions. Thus, AI should be able, if there is a PAD, to decide if we are in the situation for which the PAD can be applied, to modify the PAD if “necessary,” to add elements on patient preference when dealing with a situation not foreseen by the PAD. If there is no PAD, AI should be able to add elements on patient's preference when dealing with a medical situation.

#### 2.2.2. “WHO”—In the Contractual Relationship

**Figure 2:**
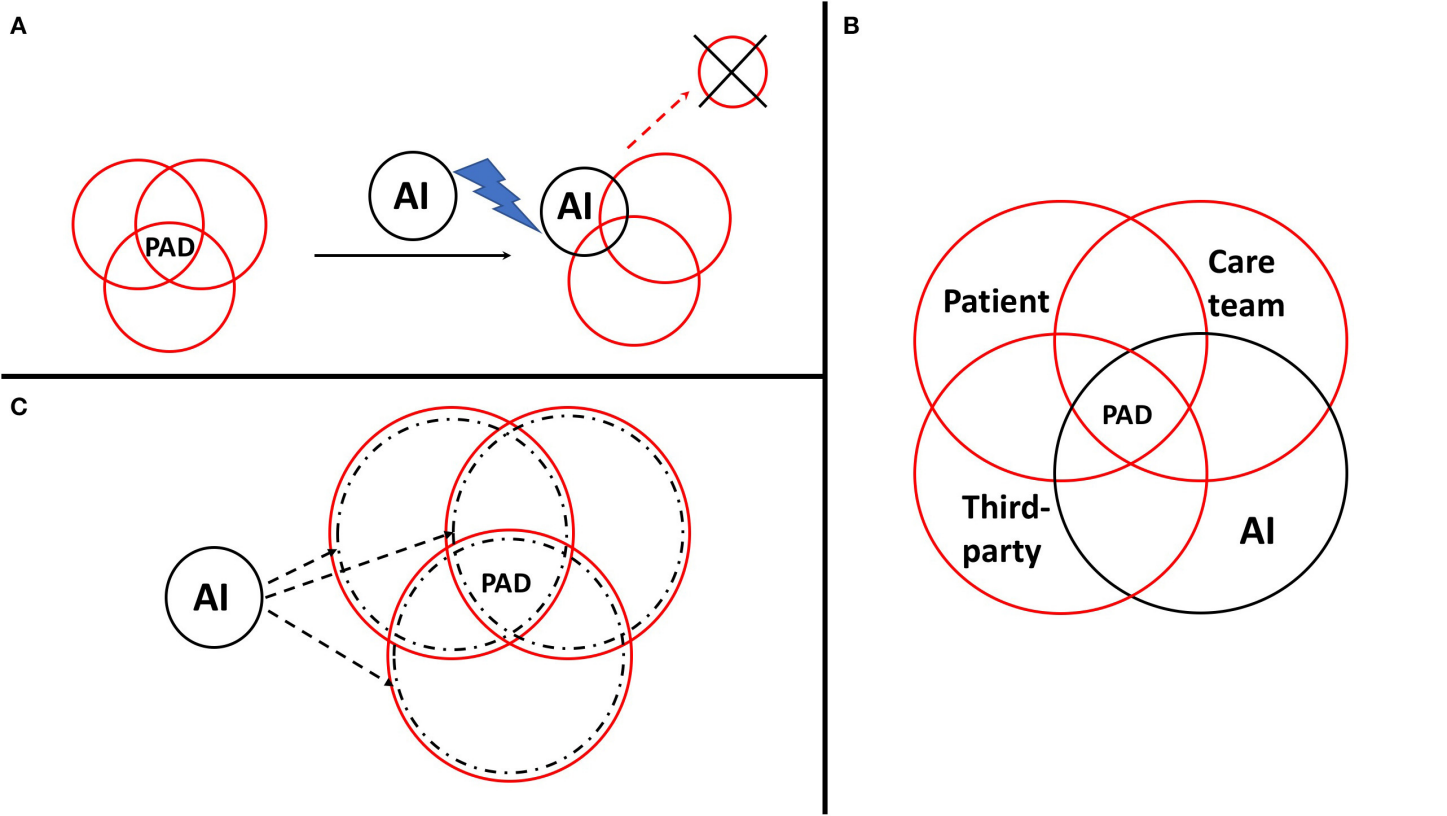
Potential places of artificial intelligence (AI) in the contractual relationship during the psychiatric advance directive (PAD) drafting process. PADs can be considered as a third-party contract in a tripartite relationship between the patient, the care team, and a third-party such as the family or the person of trust. AI could act as a substitute of a party **(A)**, as a fourth-party **(B)**, or as a partner to each party **(C)**.

In psychiatry, the family is most of the time involved in clinical decisions, and thus we can consider PADs as a third-party contract already in a tripartite relationship. When drafting PADs, it is relevant to discuss the place given to the AI, its degree of participation in the CDSS, what place the AI takes in relation to a trusted person and/or the professional if an important decision must be made (for example, stopping, maintaining, and changing treatment), and finally who makes the final decision. AI could therefore act (Figure 2).

- *As a substitute of a party of the contract*: This option is not ethical as will be seen later.
- *As a fourth party*: The option of treating AI as a “party” raises the issue of creating a juridical personality status for AI, currently being debated at the European Parliament under the concept of “electronic personality.” It could impose a responsibility for AI (inducing the creation of insurance funds by developers or users) to ensure potential victims for damages “attributable” to AI. Nonetheless, the risk is to disempower AI users (in our case the patient, the medical doctor or the third party) but also developers. In addition, applying human rights like autonomy or citizenship to an “electronic personality” raises ethical issues.
- *As a partner to each party*: An entity infiltrating each of the three parties of the contract without being a juridical personality in itself: the idea is a partnership between AI and each party (DSS for the patient or the third party, and CDSS for the clinician).

Although AIs today have no intention to harm, we can legitimately ask ourselves what will happen in several decades' time. A nonhuman decision may therefore seem very relevant at the individual level and not at all at the level of a human group and vice versa. For example, the choice not to treat a patient according to different parameters (age, symptoms, cost of treatment, rarity of treatment) may be relevant at the level of a group (significant financial loss) but may not go in the direction of maximum preservation of a patient's life span and autonomy.

It is possible that the presence of AI does not promote respect for the patient's choice but could be for AI the subject of a trade-off between the value of individual life and the preservation of collective imperatives. These aspects raise countless ethical questions about the interests of the patient and a human group. Computational ethics (36–38) raise the question of the applicability of programming ethical principles within AI. Without an overall reflection on the integration of technological systems such as AIs into the drafting of PADs, which preserve the interest of a particular person, this will result in a potential incompatibility between ADs and the use of AI. We therefore have to go through and examine each ethical principle involved.

Ethics begins with applying good practice recommendations based on values, such as in medicine: autonomy, beneficence, non-maleficence, and justice. The 2017 report (39) of the French National Commission on Informatics and Liberties adds 2 founding principles of AI ethics: loyalty and vigilance.

## 3. Principles of Medical Ethics Applied to AI in the PAD Drafting Process (40)

### 3.1. Autonomy

The principle of autonomy includes various components of the subject as follows:

- *Free will* (intentionality) was theorized by the philosophy of mind and top-down bottom-up approaches of cognition processes. If there is a disagreement between contract parties, AI could turn in favor of one of the parties (alliance) and endanger the free will of the other.
- *Free action* postulates that the act is not controlled by an external intelligent entity, conscious or artificial. It is subject to cognitive biases, manipulation, or conditioning. Free action is by definition affected by the irruption of AI in the PAD drafting process.
- *Comprehension and adaptation abilities* echo the ethical principles of AI that are loyalty and vigilance (see later). The simplification of algorithms is necessary to get the result in a reasonable amount of time for the caregiving, but also because the user should be able to understand how they work. This simplification implies the risk of loss of information or accuracy. AI cannot be involved in PADs without the patient being able to understand how it works. Hence, there is a need for patient education and specific training of AI teachers, even more specific to the population of psychiatric patients. Naturally, psychoeducation programs should include information on PADs as a prerequisite to understand AI implementation in this process.
- *The principle of dignity* includes respect for autonomy but is a broader concept that implies, in medical care, various requirements:
  - The collection of consent to care (Hippocratic Oath) is the reason why ADs were created for.
  - Valid therapeutic choices are at the cross-section of the clinician experience, the state of the scientific art, and the patient's preference. AI raises the issue of the difficult integration of “tacit” data that are considered by the clinician without him/her knowing it, i.e., heuristics that are difficult to express or objectify. Regarding the state of the art, so much the methodology (41–48) as the supremacy of science (49, 50) can be contested. In medicine, the deep learning possesses the obvious asset of rapidly finding strong correlations over an important quantity of data that cannot be analyzed by the human brain. Nevertheless, besides the high cost of constructing this “big data,” it implies significant risks that increase along with the size and efficiency of data collection: loss of oral information (that could contain important medical information) and the issue of causality in data correlations: confounding factors may come into play, hiding a useful correlation into a sum of irrelevant ones, making deep learning far from heuristic learning and reasoning of clinicians. Finally, the concept of “validity” of a therapeutic choice is subjective and depends on the patient's belief. To respect the patient's dignity would then be more about respecting the patient's beliefs, even though it may be in contradiction with evidence-based medicine.
  *- Respect for privacy and medical confidentiality* is no longer the paradigm in the field of healthcare, sharing information between the various caregivers (medical, paramedical, social workers, administrative) being now considered more beneficial for the patient. For example, in France such enlargement of the right to share medical information has been made possible in 2016 (L1110-4 of the FPHC). Confidentiality is thus gradually being replaced by professional integrity (51–53). With the generalization of electronic health records, caregivers can access the shared medical record of a patient without actually taking care of this patient: their integrity prevents them to access the record. Today, the tendency in psychiatry is to write only factual elements in the patient digital files. When invoking AI, confidentiality can only be partial, since AI skills are acquired mainly through deep learning, which uses anonymized digital records that are never completely anonymized. Indeed, patients stay traceable despite the anonymization: it is easy to “de-anonymize” data by cross-referencing specific data. These data can thus no longer be considered anonymous, which raises the issues of data ownership, control, organization, marketing, right to deletion, and specific uses such as risk assessment by insurances. Another issue is the potential lack of data precision or reliability for the determination of a probability *a priori*: the more the AI will be relevant (i.e., the more the inferred preference will be close to patient preference), the more it will have to “know” patients, including their private life. The risk is to know patients “better than themselves” and therefore their preference (conscious or unconscious) better than themselves. The AI could turn to play a role equivalent to that of the person of trust with whom private information is shared (L.1111-6 of the FPHC). Finally, if data are collected by AI, it is subject to risks such as hacking, breakdowns, and data protection system would have to be strengthened.

### 3.2. Beneficience

Concerning AI, psychiatrists are very careful on this principle (54). As it contains two elements:

- *The benefit* can be seen as an improvement of “wellness.” AI could turn to subjectively make wellness like in other societal fields where wellness becomes, through positive thinking, a moral imperative ultimately leading to malaise (55). This effect could increase within the specific population of patients suffering from psychiatric disorders. Thus, there is a need to find more objective and specific indicators to measure the benefit of a treatment. In the case of PADs, these could be the degree of adequacy *a posteriori* between the patient and the treatment received during a state of crisis.
- *The benefit-to-risk ratio* is also difficult to evaluate in the case of new technologies, risk estimations being often wrong in this field (56). Thus, the principle of objective vigilance has to be applied.

### 3.3. Non-maleficience

*Non-maleficence* invokes the idea of malaise. AI could be maleficent at every level in the PADs process. An inventory of all possible failures will be necessary, this inventory being updated along with the use of the AI, respecting the principle of objective vigilance.

### 3.4. Justice

*Justice* implies equality of care for all patients (non-discrimination), with specific adaptation to each individuality (positive discrimination) due to incompressible inequalities (genetic, developmental, or environmental). The major risk is that the specific care could get to be in function of the societal participation of the patient, marginalizing even more psychiatric patients, already undertreated on the somatic level (57–62). Encouraging clinicians to have a subjective approach also goes against harmonization of practices.

## 4. Principles of AI Ethics Applied to Medicine in the PAD Drafting Process

### 4.1. Loyalty

The principle of loyalty of AI condenses the laws of robotics proposed by (63) and the laws of algorithms (64). The AI must be a partner of the patient, particularly in the PAD drafting process, this being only possible if it respects several principles:

- *Neutrality* means treating all information equally.
- *Diversity* implies not prioritizing the answer.
- *Transparency* implies making the code available to the public. But this remains insufficient if patients are not trained to understand it.
- *Equity* involves not differentiating subjects.
- *Loyalty* means responding to what is asked.
- *Comprehensibility* must be reached by focusing on three axes (21):
  - Simplification of models, with the risks of approximation that entails.
  - Simplification of user interfaces: reduction of learning biases.
  - Education on cognitive mechanisms at work, their strengths and weaknesses, and in particular the principle of precaution against the high risk of digital divide with the psychiatric population. Indeed, if users are not formed well enough, the learning bias leads to a misuse of AI systems. Education comprises formation of AI developers, clinicians, patients, families, and the development of tools for the management of personal data and data processing by the patients themselves.

### 4.2. Vigilance (Also Referred as Reflexivity or Auditability)

Vigilance makes it possible to cover the risks inherent to new technologies, because anticipating all situations is impossible. It is important to have standards as a basic precaution, but they have to stay flexible enough to allow technological innovation. Vigilance must be the responsibility of the state and in practice, it should be implemented through the creation of committees, recruited on referral, litigation or during automatic audits. These committees would have to be open to patients and users of healthcare systems. Vigilance is not just to reliably identify technological failures and propose alternatives to avoid them. In the absence of failures, it must also be sanitary: global audits should be implemented to assess for the success or the failure of the use of new AI technology on the physical and mental health of patients.

## 5. Conclusion and Suggested Guidelines

This reasoning on the introduction of technological devices in the PAD drafting process confronts caregivers and patients with preconceived ideas about both ADs and AI. Regarding the anticipated guidelines, some professionals are still skeptical about the introduction of a crisis plan (16): doubts about the relevance of this crisis plan, addition of documents to be taken into account, lack of consideration of the crisis plan by the teams, etc. With regard to new technologies, a number of preconceived notions also persist, both on the part of caregivers and patients (54). Concerns about AI include concerns about confidentiality and data storage, particularly when it comes to sensitive data such as health data. In this context, it would be appropriate for the data collected by these intelligent systems to comply with the legislation on the confidentiality of health data (65) as defined in the FPHC. At present, systems, such as those mentioned above, are capable of making reliable predictions based on algorithms and a large database, respecting legislation on confidentiality and the use of health data do not yet exist. In this context, we can propose several recommendations:

### 5.1. Support: AI Must Remain a Decision Support System, and Seen as a Complement to the Decision, a Partner of the Parties of the PAD Contract

It should always be subject to validation by a professional, whether it is the patient's reference professional or an expert identified and solicited through the use of a telemedicine service. The presence of technological devices such as AI helps to bring new elements (medical data, therapeutic options not considered by health professionals). In order for this input to continue to be relevant to the patient and the professional, a probability system should be in place to weight:

- The different treatment options, possible outcomes and their likely influence on symptoms (and which ones).
- Ecological data (data reported to the professional), their potential evolution, and impact on the patient's quality of life.

### 5.2. Choice: It Must Be Let to Patients Whether They Wish to Use AI or Not, Which Type of AI, at What Step in the PAD Process

Patients must be able to be systematically informed of the use of an intelligent technological device during their care journey and give their consent for its use and authorization on the different types of data collected. Patients must be able to choose, at each level, whether they consent to the use of AI for data collection: for which data precisely do they consent, and for which use (collection, sharing of information with the professional, CDSS, research, etc.).

### 5.3. Information: Make AI Understandable

A significant amount of information and education work remains to be accomplished on the functioning and relevance of intelligent systems. It will also be essential to explain the limitations of such tools, including the degree of feedback in the event of a system failure. For this purpose, massive open online courses, workgroups, serious games could be flexible tools. It is primordial to evaluate the level of comprehension, perception, and acceptability of these educational tools, with the use of experimental studies. A final checklist with items verifying the comprehension of the process would also be mandatory. This information should be subject to strengthened provisions in the case of vulnerable persons in order to ensure that free and informed consent is obtained.

### 5.4. Vigilance: Create a Committee That Will Audit These New Tools in Terms of Successes and Failures, Security, and Relevance

In addition, a set of feedback systems must be provided for in the event of errors (a system of probability or reliability of therapeutic options, detection of errors by the system itself, clinical sense) integrating targeted and random control systems on the different functionalities offered by the AI. In order to allow for the integration and optimal use of these systems within healthcare services, it will be necessary to create new legal frameworks for the use and regulation of these systems and the data obtained through these systems. In particular, regulation must consider the level of sensitivity of the health data collected and their impact on medical decisions. In addition, the use of new technologies must respect the rules and ethical principles of caregivers. In fact, it will be necessary to support health professionals in the use of new technologies that respect these rules inherent to their profession. One of the ways in which these tools could be deployed is to implement them gradually with feedback (evaluation and research on their relevance in healthcare), ethical considerations on new technologies and finally anticipation of new cases of use. The subject of PADs raises more than any other the delicate balance between support for innovation and the necessary ethical regulation. Current issues related to new technologies give rise to important debates on the impact on the maintenance of financial and human resources, the quality of care, the preservation of the human link between caregiver and patient, and respect for patients' rights.

This paper is a reflection by medical professionals on how to employ new information technology tools and techniques for the improvement of the patient's hospital experience. It is fully understood that some suggestions may be in contravention of legal dispositions of some national jurisdictions and that special permission or even legislation may be required to eventually put them into practice in the future.

## Author Contributions

SM, VA, and CF-S contributed to the conceptual framework and the writing of this work. SM supervised the development of this work. OB, RM, BM, C-SP, and FF contributed to the manuscript evaluation and reviewing. AB and FF contributed to the editing. All authors contributed to the article and approved the submitted version.

## Conflict of Interest

The authors declare that the research was conducted in the absence of any commercial or financial relationships that could be construed as a potential conflict of interest.
